# Lyophilization to enable distribution of ChAdOx1 and ChAdOx2 adenovirus-vectored vaccines without refrigeration

**DOI:** 10.1038/s41541-023-00674-2

**Published:** 2023-06-05

**Authors:** Cheng Zhang, Adam Berg, Carina C. D. Joe, Paul A. Dalby, Alexander D. Douglas

**Affiliations:** 1 Department of Biochemical Engineering, University College London, Bernard Katz Building, Gower Street, London, WC1E 6BT United Kingdom; 2 Jenner Institute, University of Oxford, Old Road Campus Research Building, Roosevelt Drive, OX3 7DQ Oxford, United Kingdom

**Keywords:** Recombinant vaccine, Pharmaceutics

## Abstract

Distribution of vaccines which require refrigerated or frozen storage can be challenging and expensive. The adenovirus vector platform has been widely used for COVID-19 vaccines while several further candidate vaccines using the platform are in clinical development. In current liquid formulations, adenoviruses require distribution at 2–8 °C. The development of formulations suitable for ambient temperature distribution would be advantageous. Previous peer-reviewed reports of adenovirus lyophilization are relatively limited. Here, we report the development of a formulation and process for lyophilization of simian adenovirus-vectored vaccines based on the ChAdOx1 platform. We describe the iterative selection of excipients using a design of experiments approach, and iterative cycle improvement to achieve both preservation of potency and satisfactory cake appearance. The resulting method achieved in-process infectivity titre loss of around 50%. After drying, there was negligible further loss over a month at 30 °C. Around 30% of the predrying infectivity remained after a month at 45 °C. This performance is likely to be suitable for ‘last leg’ distribution at ambient temperature. This work may also facilitate the development of other product presentations using dried simian adenovirus-vectored vaccines.

## Introduction

The COVID-19 pandemic has highlighted the challenges of the distribution of vaccines which require refrigeration, or in some cases ultra-cold transport^1^. In higher-income countries, cold chain requirements have also been key determinants of vaccine programme design, including which COVID-19 vaccines were offered to which populations^2^. In lower-income countries, cold chain requirements have often determined whether or not a particular COVID-19 vaccine can realistically be distributed at all^3,4^. The relatively short shelf-lives of some products, due to concern about potency loss at 2–8 °C, have resulted in millions of doses of vaccine being wasted^5^. The cost of maintenance of a 2–8 °C cold chain is significant, and likely to be significantly higher for products requiring ultra-cold transportation.

The pandemic has also highlighted the value of vaccine platform technologies which are applicable to multiple diseases, and can be rapidly adapted in response to emerging pathogens. The relative stability of adenovirus vectors is one of the platform’s key advantages, as compared to mRNA vaccines. More than 3 billion doses of adenovirus-vectored vaccines have been distributed to date, typically with 6–9-month shelf-lives in the liquid formulation at 2–8 °C. This stability is particularly relevant in resource-poor contexts and in facilitating vaccination outside traditional healthcare settings. Nonetheless, it would be highly advantageous if these vaccines could be distributed outside the cold chain and/or their relatively short shelf-lives could be extended.

The versatility of vaccine platform technologies enhances the value of formulation improvements, as they can potentially be applied to vaccines against multiple diseases. To our knowledge, all licensed adenovirus-vectored vaccines have been distributed in liquid formulation. As far as we are aware, no liquid formulation has demonstrated potential for clinically-relevant concentrations of adenovirus to be stored at 20 °C for more than a few days^6–8^. Some studies have reported greater stability in liquid formulation, but have used concentrations of vaccine at least one order of magnitude lower than those typically used clinically (c. 1×10^11^ VP/mL)^9,10^.

Lyophilised formulations have been used for many years for human and veterinary live-attenuated adenoviral vaccines, but these products nonetheless typically require storage at 2–8 °C^11,12^.

The peer-reviewed literature discloses formulations and cycle parameters that achieve stability of adenovirus-vectored vaccines at temperatures up to 37 °C for periods of around one month^13–15^. Early work by Croyle and colleagues showed that lyophilization of human adenovirus serotype 5 (AdHu5) formulations combining sucrose, mannitol and Pluronic F68 could be accomplished with in-process loss of ≤50% of infectivity (≤0.3 log10-fold), followed by storage with the negligible further loss for one month at room temperature, but did not explore higher temperatures^13,14^. Subsequently, a formulation combining inulin and mannitol was claimed to achieve cumulative loss after lyophilization and 1 month of storage at 37 °C of c. 50% of infectivity (≤0.3 log10-fold)^15^.

Further data are reported in the patent literature and in publications that do not fully disclose the details of the excipient composition or provide limited information about the preservation of potency at elevated temperatures^16–18^. Notably Drew and colleagues patented a lyophilization method using specific excipients (trimethylglycine and dimethylsulfone), which they reported to achieve loss of c. 0.5 log10-fold over 15 weeks at 37 °C^17^.

Licensed adenovirus-vectored vaccines have used three serotypes: AdHu5 (Cansino’s COVID-19 and Ebola vaccines); the human serotype Ad26 (Johnson & Johnson’s COVID-19 and Ebola vaccines); and ChAdOx1, a species E simian adenovirus (the Oxford / AstraZeneca COVID-19 vaccine). All of the studies cited above regarding adenovirus vectored-vaccine lyophilization used viruses from within species group C (AdHu5, a commonly used model serotype, or the simian adenoviruses ChAd3 and ChAd155)^19^. Although our experience is that the stability of different adenovirus-vectored serotypes in a given formulation is quite similar, there is relatively little published data relating to lyophilization of species E simian adenoviruses, such as the ChAdOx1 vector used for the Oxford / AstraZeneca COVID-19 vaccine.

We previously evaluated the stability of adenovirus-vectored vaccines based on two species E serotypes (ChAdOx1 and ChAdOx2, which are derived from the Y25 and AdC68 serotypes, respectively^20,21^) using the inulin and mannitol-containing lyophilized formulation developed by Chen and colleagues^8,15^. We observed quite significant in-process loss and run-to-run variation: 75–90% of the infectious titre of the product was lost during the lyophilization process, and stability was insufficient for long-term storage in an uncontrolled environment^8^.

Here, we report the development of an optimized formulation and a suitable process for the lyophilization of ChAdOx1 vectors. We focus primarily upon the preservation of viral infectivity, which is the principal indicator of potency used to define adenovirus-vectored vaccine shelf life^22,23^. Throughout this study, the virus was used at clinically-relevant concentrations (c. 1×10^11^ genome containing virus particles/mL, unless stated otherwise), and loss of infectivity, measured using the luciferase assay, is reported as ‘log10-fold’, i.e. change in the log10-transformed infectivity titre.

## Results

### Selection of excipients

We initially sought to screen a wide range of potential formulations using a ‘design of experiments’ approach. To facilitate screening, we used our previously-established approach of lyophilization in microplates^24,25^, and an infectivity assay based upon measurement of a virus-expressed luciferase transgene. This assay provided a broad linear range, correlation with the gold-standard hexon immunostaining assay, and low between-assay variability (Supplementary Fig. 1), and allowed substantially greater throughput than hexon immunostaining or FACS-based infectivity assays. This was typically used to calculate ‘in-process’ infectivity loss occurring during drying and ‘in-storage’ loss after thermal challenge (typically 30 days at 30 °C). In some cases in-process loss was measured after a further 30 days at 4 °C, making an assumption of minimal further loss over this period (and providing a conservative estimate if this assumption was inaccurate).

**Fig. 1:**
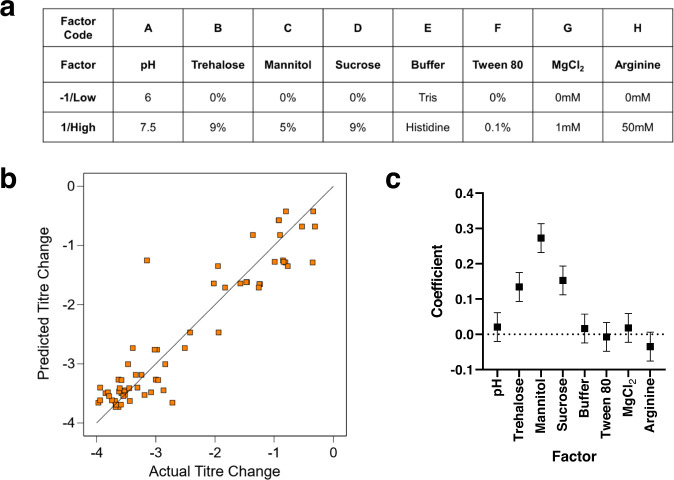
Trehalose, sucrose and mannitol enhance product stability. **a** An eight-factor two-level partial factorial design was used to screen lyophilisation formulations to identify factors that improve stability of ChAdOx1 luciferase after 1 month of storage at 30 °C. Samples were dried using cycle A (Supplementary Data, sheet 2) in Virtis Advantage Plus. Study was performed twice and mean results taken for analysis. **b** Model goodness-of-fit: comparison of model-predicted and actual changes in infectivity titre. **c** Modelled main-effect coefficients for the eight factors. Positive values indicate stability improvement by the presence of the excipient. Error bars show 95% confidence intervals.

We initially explored a panel of excipients (trehalose, sucrose, mannitol, polysorbate 80, arginine and MgCl_2_) which have previously been reported to give favourable results in lyophilization of adenoviruses or other biologicals, and pH buffering to 6 or 7.5 using either histidine or Tris buffer (noting that pH 6 is outside the optimal buffering range of Tris). In a 1/8 fractional factorial design, we evaluated these eight factors, each at two levels (Fig. 1a). For this and all subsequent experiments, full details of each formulation are provided in Supplementary Data sheet 1, and lyophilization cycle parameters are provided in Supplementary Data sheet 2. Mannitol, sucrose and trehalose were found to have significant positive effects upon titre after 30 days of storage at 30 °C (Fig. 1c). The best-performing formulation achieved total loss in-process plus in-storage over one month at 30 °C of 0.4 log_10_-fold (range 0.3–0.5, mean of two experiments, one of which included two technical replicates). This compared favourably to mean loss of 1.2 log_10_ fold (range 1.0–1.5, replication as stated previously) in an inulin/mannitol-based formulation reported by Chen et al.^15^, where in-process loss was not measured separately.

**Fig. 2:**
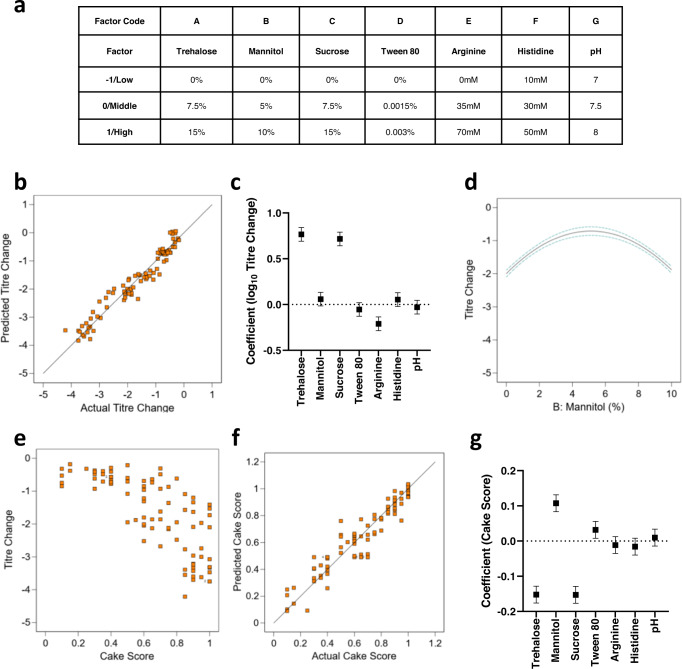
15% trehalose, 15% sucrose, 5% mannitol formulations result in good stability but poor cake morphology. **a** A seven-factor three-level partial factorial design was used to screen lyophilization formulations. Higher concentrations of sucrose, trehalose and mannitol were used based upon previous results. Formulations were dried using cycle A. After 1 month storage at 30 °C cake quality was scored and titre loss measured by luciferase quantification. Samples were dried using cycle A (Supplementary Data, sheet 2) in Lyostar 3. **b** Goodness of fit comparison for model predicted and actual changes in infectivity titre. **c** Modelled main effect coefficients for titre change for the seven factors. Error bars show 95% confidence intervals. **d** Effect plot for mannitol % on titre change. Dashed lines show 95% confidence intervals. **e** Relationship between titre change and cake score. **f** Goodness of fit comparison for model predicted and actual changes in cake score. **g** Modelled main effect coefficients for cake score for the seven factors. Error bars indicate 95% confidence intervals.

We proceeded to a second optimisation screen in microplates, in which seven factors were each tested at three levels in a central composite design (Fig. 2). This included higher concentrations of trehalose, sucrose and mannitol, the factors which had the greatest positive effects in the first screen. A model fitted to loss of infectivity titre after drying followed by 30 days of storage at 30 °C demonstrated significant positive effects of both sucrose and trehalose extending to the upper tested concentrations (15% w/v of each), but that increasing mannitol beyond the previously tested 5% was detrimental (Fig. 2c). Arginine appeared detrimental while no other factor had a significant effect.

We noted that cake morphology was highly variable in these experiments. To assess the effect of excipients upon cake morphology, the appearance of cakes from the second screen was scored against an in-house scale (Supplementary Fig. 2) by an observer blinded to the formulation in each well. It was apparent that there was an inverse relationship between cake quality and product stability (Fig. 2e). Consistent with this, a model fitted to the cake morphology data (Fig. 2f, g) demonstrated detrimental effects of high trehalose and sucrose concentrations, and a U-shaped effect of mannitol, the inverse effects of these factors upon titre after thermal challenge.

We attempted to explore further increases in trehalose and sucrose concentrations, again using the microplate format. This resulted in further cake deterioration, to the point that titre could not be analysed because of adherence of cake to the wells’ walls and lids. We also tested the effect of addition of further excipients, including dextran, PVA, glycine and serine, with the aim of improving the cake appearance while retaining the titre. However, these did not improve the cake appearance, and some had an adverse effect upon titre (Supplementary Fig. 3).

To gain insight into the mechanisms of infectivity loss in poor-performing formulations, we measured a range of biophysical parameters in vaccine which had been reconstituted after lyophilization and storage for a month at 30 °C. We selected nine formulations for these studies, spanning the range of stabilization performance observed in the screening (three good, three intermediate and three poor). For each of these formulations, we measured particle size (by dynamic light scattering, DLS); total vector concentration (by UV spectrophotometry after SDS treatment); and intrinsic fluorescence during a temperature ramp (using a UNit instrument [Unchained Laboratories Inc]). In formulations which had preserved viral titre poorly after thermal challenge in our screening studies, we observed increased particle size, decreased barycentric mean fluorescence wavelength (possibly suggesting burying of tryptophan residues), with a decrease in vector concentration which was modest by comparison to the loss of infectivity (Supplementary Fig. 4). These changes would be consistent with virus aggregation as a mechanism of potency loss, and suggest that rapid biophysical assays might have a role in rapid initial ‘rule-out’ screening of poor-performing candidate formulations for adenovirus stabilisation.

Based upon the model-predicted optimal formulations in the preceding work, we prepared a formulation comprising 15% Trehalose, 15% Sucrose, 5.45% Mannitol, 0.003% Tween 80, 50 mM Histidine pH 7. This formulation is henceforth referred to as TSM2. We proceeded to validate the performance of this formulation in lyophilization in vials (rather than microplates). We performed two experiments, one of which included three different vaccines.

**Fig. 3:**
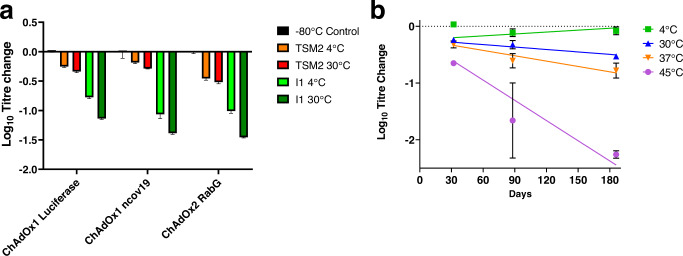
Performance of trehalose/sucrose/mannitol formulation after drying in via*ls*. **a** Titre change of three different viruses, measured by hexon immunostaining assay, at 4 °C or 30 °C after 32 days. Samples had been dried using Cycle A in formulations TSM2 and I1. Samples were dried using cycle A (Supplementary Data, sheet 2) in Virtis Advantage Plus. Values are the mean of 3 replicates with range shown by error bars. **b** Loss of titre for ChAdOx1 luciferase (measured by luciferase quantification) dried in formulation TSM2 using cycle A and stored at 4 °C, 30 °C, 37 °C and 45 °C for 1, 3 and 6 months. Lines shown were fitted by linear regression using Prism 9.0 (GraphPad), under the constraint that all should share the same Y-intercept (signifying the in-process loss). Samples were dried using Cycle A (Supplementary Data, sheet 2) in Virtis Advantage Plus. Points show the mean of three replicates with range shown by error bars.

The first of these experiments (shown in Fig. 3a) sought to corroborate our initial results using luminescence-based measurement of the infectivity of ChAdOx1 luciferase. We therefore determined infectivity titre with the widely-used hexon immunostaining assay, and included as a comparator a formulation known as I1 which has previously been shown to achieve good results in adenovirus stabilisation^15^. As well as ChAdOx1 luciferase, this first experiment also included ChAdOx1 nCoV-19 (as used in Vaxzevria, the licensed ‘Oxford-AstraZeneca’ COVID-19 vaccine) and ChAdOx2 RabG (a candidate rabies vaccine based upon another species E simian adenovirus, which is currently in clinical trials)^26,27^. Across the three viruses, total infectivity loss of TSM2 formulations was consistently <0.5 log_10_-fold (i.e. <70%) after drying followed by one month at 30 °C, while losses in the comparator formulation I1 were consistently >1.0 log_10_-fold (i.e. >90%) (Fig. 3a).

The second experiment (shown in Fig. 3b) assessed the impact of storage of ChAdOx1 luciferase over longer periods and at higher temperatures. Total loss after drying and storage at 30 °C for 6 months was 0.53 log_10_-fold (70%). The half-time of in-storage infectivity decay at 45 °C was 26 days.

### Iterative modification of cycle parameters to achieve satisfactory cake morphology and stability

Having identified formulations with encouraging stabilization performance (but noted that the best formulations in terms of stability had unsatisfactory cake appearance) we proceeded to investigate whether we could modify the freezing and drying process to improve cake appearance of these promising formulations, while retaining their stabilization performance.

Some lyophilization cycles use an annealing step, in which the temperature is elevated to a level between the glass transition temperature (T_g_’) of the freeze-concentrated amorphous phase and the melting temperature of the crystalline phase. After several hours at this elevated temperature, the product is returned to lower temperature and vacuum applied to begin primary drying. This allows the growth of crystals (in the case of our formulation, potentially of ice and/or mannitol) by Ostwald ripening. The presence of large crystals of solute can support the cake (reducing collapse), while large ice crystals can result in a more porous cake and enhance primary drying^28,29^. Limited non-peer-reviewed data suggests encouraging results of annealing during lyophilization of adenovirus-vectored vaccines in a trehalose-containing formulation fairly similar to ours^18^.

To identify the temperature window for an annealing step using formulation TSM2, we initially characterised the glass transition temperature of the freeze-concentrated solute (T_g_’) and the melting temperature (T_melt_) of the formulation predicted to achieve optimal stability (by the design of experiments models fitted to earlier data). Temperature-dependent impedance measurement using a Lyotherm instrument (Biopharma Group) and differential scanning calorimetry (DSC) provided similar estimates of −35 °C for T_g_’. DSC measured T_melt_ as −27 °C. T_g_’ and T_melt_ were also measured for 7 other formulations, selected to represent the range of performance seen in the earlier work.

We proceeded to explore the effect of modified cycle parameters, incorporating an annealing step and seeking to improve cake morphology while retaining the product stability achieved in the earlier work. We investigated the performance of two alternative cycles (Cycles B and C, shown in Supplementary Data Sheet 2), each of which included an annealing step. Cycle C also had the primary drying time prolonged until comparison of Pirani and capacitance manometer measurements indicated no further water removal. In each of these cycles, we tested the same range of formulations, which were selected for the biophysical study above, drying in microplates to increase throughput. Due to variation of T_g_’ and T_melt_ between the formulations, it was not possible to select a single temperature lying between these critical temperatures for all formulations. We initially chose −28 °C as the annealing temperature as it was 4 °C higher than the highest T_g_’ (−32.3 °C) and at the lower end of the range of T_melt_ values. As shown in Supplementary Fig. 5, these cycle modifications improved the cake morphology.

We next sought to assess the performance of a cycle incorporating annealing when drying in vials rather than microplates (Cycle D, Supplementary Data Sheet 2). For this, we selected a lower annealing temperature of −30 °C, due to the relatively low T_melt_ of the best-stabilising formulation (−27.0 °C). Product stability at 30 °C was fairly similar to that in microplates but, although there was some improvement in cake morphology, cake appearance in vials remained sub-optimal with a ‘skin’ lying over partially collapsed cake, and greater heterogeneity of appearance than had been apparent in microplates (Supplementary Fig. 5).

To identify the stage of the cycle at which this heterogeneity was arising, we used time-lapse photography to image vials during drying using the ‘Cycle D’ recipe shown in Supplementary Data Sheet 2. Time-lapse images revealed that in some, but not all, vials, distortion of the surface of the cake occurred suddenly (over a period of seconds) around 1 h after the shelf temperature was raised from −30 °C to 0 °C, along with Pirani *vs* capacitance manometer data suggesting accelerated sublimation. This would be consistent with the increased temperature resulting in a build-up of vapour under the surface of cakes with low porosity, and leading to explosive release. We therefore hypothesized that slower temperature increase during drying would minimise this effect. A cycle incorporating a gradual temperature ramp (‘Cycle E’ recipe shown in Supplementary Data Sheet 2) did indeed result in more consistent and satisfactory cake appearance (an example from a subsequent experiment is shown in Supplementary Fig. 6A).

We further hypothesized that if a ‘skin’ at the cake surface was indeed affecting cake morphology, there may be a relationship between filling depth and cake morphology. Up to this point, for work in vials we had used a filling volume of 650 μL and virus particle concentration of c. 1×10^11^ VP/mL (matching the volume and titre of liquid formulation in a vial providing a single human dose). If reconstituted in the same volume, however, this would result in markedly hypertonic solution unsuitable for intramuscular vaccination. We therefore proceeded to evaluate the influence of reduced filling depth and increased virus concentration upon product stability and cake morphology (Supplementary Fig. 6A). Drying a human dose of 6.5×10^10^ VP in 200 μL of formulation JL2 at a product concentration of 3.25×10^11^ VP/mL, followed by reconstitution in 650 μL of water, would result in a human dose in an extractable volume of 500 μL at an acceptable tonicity of 400 mOsm/L.

With reduced filling volumes, including in the vials filled with 200 μL at 3.25×10^11^ VP/mL, we observed satisfactory cake appearance and improved product stability over 50 days at 30 °C (Supplementary Fig. 6). Some cake deterioration was noted in storage at elevated temperature (Supplementary Fig. 6C).

### Effect of trehalose/sucrose ratio, annealing and extended secondary drying on stability and cake appearance at multiple temperatures

Informed by the work described above, we proceeded to evaluate within a single experiment the effect of annealing and extended secondary drying upon formulations similar to the previously-identified optimum. Within the same experiment, we sought to further define the optimal buffer composition by comparing formulations containing various ratios of trehalose and sucrose (totalling a constant 30% w/v), and also varying histidine concentrations. Mannitol and PS-80 concentrations were held constant at 5.45% w/v and 0.003%, respectively. We used vials containing clinically-relevant vaccine doses of 5×10^10^ VP and formulations / fill volumes suitable for reconstitution to provide a human dose with acceptable tonicity for intramuscular injection, as above.

**Fig. 4:**
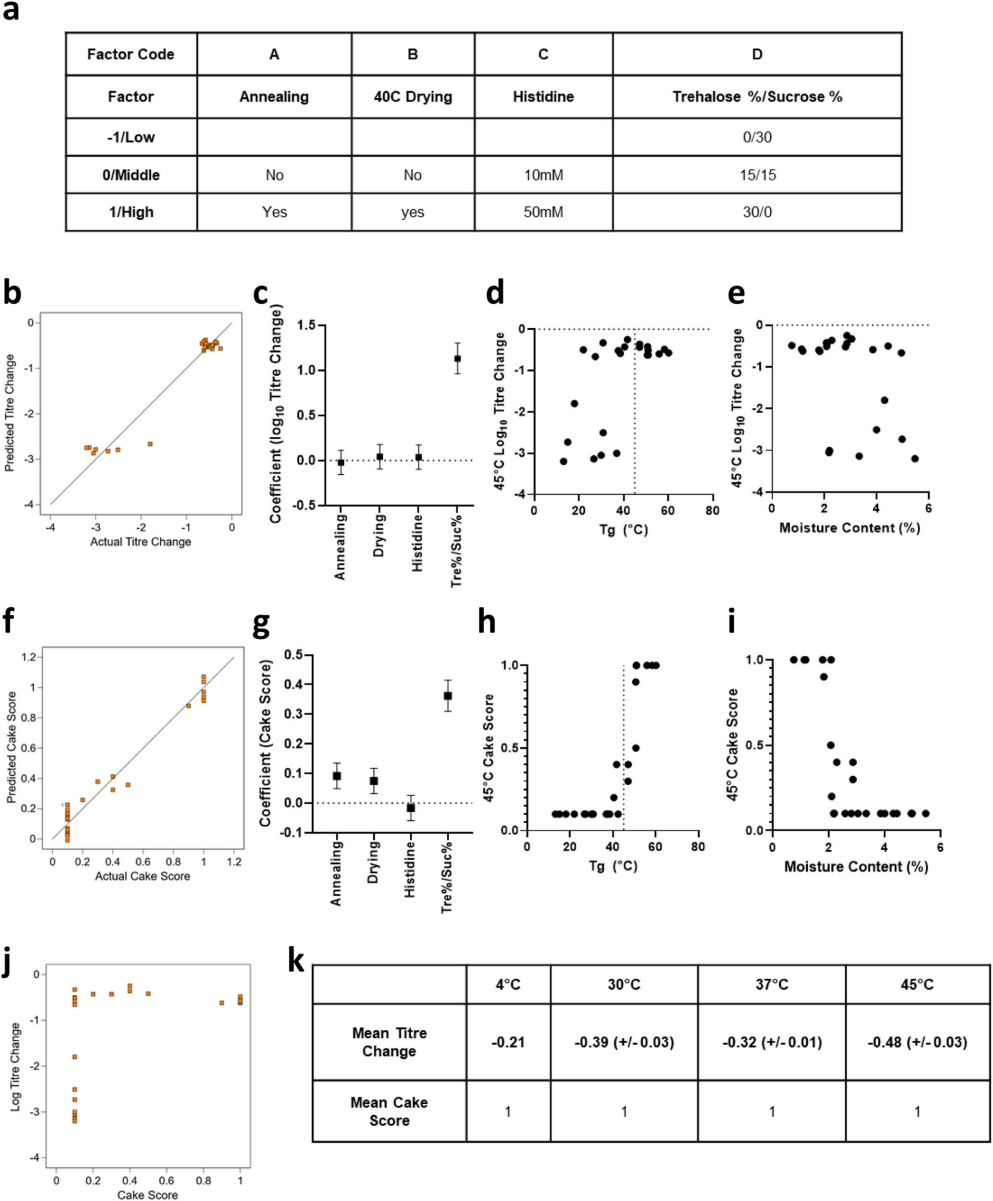
Testing effects of drying cycle changes, histidine concentration and ratio of trehalose/sucrose on cake appearance and stability. **a** A four-factor multi-level design was used to screen both formulation and drying process for factors that improve stability of ChAdOx1 luciferase after 1 month of storage at 45 °C. Samples were dried using variations of Cycle F (Supplementary data sheet 2) in Lyostar 3. Subsequent panels show the mean of duplicate vials for each formulation in each assay, with the exception of residual moisture which was measured in singlicate. **b** Model goodness of-fit for titre change: comparison of model-predicted and actual changes in infectivity tire. **c** Modelled main effect coefficients for the 4 factors for infectivity titre, positive values indicate stability improvement. Error bars show 95% confidence intervals. **d** Relationship of T_g_ (measured by DSC on dried solids) with infectivity titre. **e** Moisture content % vs infectivity titre. Moisture content measured by Karl Fischer. **f** Model goodness of-fit for cake score: comparison of model-predicted and observed cake scores. **g** Modelled main effect coefficient for cake score. Positive values indicate improvement in cake appearance. Error bars show 95% confidence intervals. **h** T_g_ vs cake score. **i** Moisture content % vs cake score. **j** Cake score vs titre change. **k** Performance of best formulation and cycle (30% trehalose, 0% sucrose, 10 mM histidine, pH 7, 5.45% mannitol, 0.003% Tween 80, with annealing and 40 °C drying).

We evaluated the effect of these variables using a full factorial design to assess the impact on multiple outcome variables encompassing stability of potency and appearance at multiple temperatures from 4 to 45 °C, as well as physical characteristics of the dried solids (residual moisture analysis and T_g_ measurement by DSC) (Fig. 4).

Comparing conditions, it was apparent that formulations containing trehalose had a beneficial effect upon the stability of both potency and cake appearance at higher temperatures, with the 30% trehalose / 0% sucrose formulation particularly beneficial for cake stability at 45 °C. Histidine concentration had no significant effect on any endpoint. Annealing and extended secondary drying each had a beneficial effect upon the stability of both potency and cake appearance at higher temperatures but this was less apparent or insignificant in 30% trehalose / 0% sucrose formulations.

Residual moisture content and T_g_ were correlated with stability of potency and cake appearance (Fig. 4). As expected, T_g_ was highest for the 30% trehalose / 0% sucrose formulations. Conditions achieving good cake stability tended to have moisture content below 2% and T_g_ above 45 °C. Similar but weaker relationships were observed between stability of infectivity, residual moisture, and T_g_.

Across the 8 conditions including ≥15% trehalose and annealing, infectivity stabilisation was very similar to the earlier work with formulation TSM2, with low loss in-process (median 0.24 log_10_-fold) and good stability over a period of one month at temperatures of up to 45 °C (median 0.23 log_10_-fold post-process additional loss).

Stability was excellent over one month at 30 °C. Across the 16 conditions, including ≥15% trehalose, median titre of samples stored at 30 °C for one month was not biologically or statistically significantly lower than matched samples stored at 4 °C (mean difference = 0.02, 95% confidence interval for mean −0.05–0.1, *p* = 0.54 by paired t-test).

Taken together, these results show that formulations containing trehalose and mannitol can achieve good stability of species E adenovirus infectivity. Annealing and extended secondary drying appear dispensable for the preservation of infectivity, but may have a beneficial effect upon cake appearance after storage.

## Discussion

This work has shown that lyophilization of amorphous sugar-based formulations can achieve encouraging stability of a species E simian adenovirus. Broadly, increasing concentrations of sucrose and trehalose, up to a total of 30% w/v, improved the preservation of infectivity. There was little evidence of benefit from nonsugar excipients: good performance was achieved by formulations containing only 30% trehalose and/or sucrose, 5.45% mannitol, 10 mM histidine as a buffer and 0.003% PS-80.

In the early stages of this study, formulations which improved preservation of virus potency were associated with poor appearances of the lyophilised cake. To resolve this, we initially explored selected modifications to the drying cycle: incorporation of an annealing step, and a slow increase in temperature during primary drying to avoid sudden sublimation, were found to have a beneficial effect upon cake appearance. Cycles incorporating these modifications were able to achieve good product stability and reasonable cake appearance when a realistic human dose was dried in vials in a formulation including 15% trehalose and 15% sucrose. We subsequently demonstrated that extended secondary drying further enhanced cake appearance of such formulations.

Finally, comparison of different trehalose / sucrose ratios among formulations with total disaccharide content of 30% w/v suggested that removal of sucrose (leaving 30% trehalose) had a beneficial effect upon cake appearance without having any significant effect upon the preservation of infectivity. With 30% trehalose formulations, annealing and extended secondary drying appeared unnecessary to achieve satisfactory cake appearance, suggesting such formulations may enable shorter cycle times than sucrose–trehalose mixtures. Cycles incorporating annealing still trended to have superior in-storage cake stability at 45 °C, but this effect was not apparent at lower temperatures (Supplementary Fig. 7).

We believe that the product stability reported here is favourably comparable to that achieved by previously described methods for adenovirus lyophilization^13–16^: we are not aware of a previous report of a method capable of achieving <0.5 log_10_-fold in-storage titre loss over a month at 45 °C. The approach reported here has some similarities to that previously reported in a patent held by GlaxoSmithKline (GSK), sharing the use of trehalose, reduced sodium chloride, and in some cases annealing^18^. Our approach differed from the GSK patent both in method and in results. With respect to method, we used mannitol and higher disaccharide concentrations, completely omitted sodium chloride, used lower annealing temperatures (or, in some cases, omitted annealing), and applied the method to species E simian adenoviruses (rather than species C). With respect to results, the GSK patent did not report stability under conditions more stringent than a few days at 37 °C.

Vaccine vial monitors (VVMs) are widely used to provide assurance that vaccines have been stored in suitable conditions during distribution. The most stable vaccines carry VVMs capable of indicating cumulative exposure of more than 30 or more than 250 days at 37 °C (VVM 30 and VVM 250, respectively)^30^. In addition, the WHO ‘Controlled Temperature Chain’ initiative involves the use of ‘peak threshold indicators’ which indicate exposure of more than 15 min to temperatures above 40 °C, and requires vaccine manufacturers to provide evidence of acceptable stability over a minimum of 3 days at 40 °C^31,32^. Our data obtained at 45 °C (Figs. 3 and 4) suggests the suitability of vaccines lyophilized using this method for VVM 30 indicators and for the ‘Controlled Temperature Chain’ approach. Further data obtained at 37 °C and 40 °C would be of value in confirming this.

Although the performance of this approach is encouraging and lyophilized vaccines are widely used, we note the results of the recent Vaccine Innovation Prioritisation Study (organised by a number of expert stakeholders, including the World Health Organisation and Bill and Melinda Gates Foundation). This study identified the desirability of thermostabilisation approaches which, unlike lyophilization in vials, avoid the need for manual reconstitution^33^. We hope that our work here may facilitate the development of new product presentations based upon dried product, such as dual-chamber syringe devices, powders for inhalation, and enteric-coated oral products.

## Methods

### Viruses and infectivity titration

ChAdOx1 nCoV-19, ChAdOx1 luciferase and ChAdOx2 RabG were prepared by growth on suspension-adapted HEK293 Trex cells (Thermo Fisher Scientific), detergent-mediated lysis 48 h after infection, clarification using a C0SP depth filter (Merck Millipore), and anion exchange chromatography using a SartoBind Q capsule (Sartorius)^20,34,35^. The anion exchange eluate was concentrated and buffer-exchanged into 8.6% sucrose, 8.6% trehalose w/v formulation buffer with no other excipients by tangential flow filtration using a flat sheet filter with 300 kDa molecular weight cut-off (Biomax, Merck Millipore).

For infectivity titration of ChAdOx1 luciferase, HEK293-TRex Cells (ThermoFisher) were grown in complete DMEM (10% FCS, 100 U penicillin, 0.1 mg streptomycin/ml, 5mM L-glutamine) in 96 well plates (Corning, 3300), to a confluency of 80–100%. To induce expression of the luciferase transgene, 10 µg/ml tetracycline was added to the medium prior to infection. For each sample to be titered, duplicate 25-fold serial dilutions were prepared in the same complete medium, and added to the cells, alongside a standard curve created by a series of two-fold dilutions of a sample of known infectivity. Every infectivity plate included a reference sample used to calculate titre change (difference between the unknown sample and the reference sample). This reference sample was taken from the stock to be dried and frozen at −80 °C in A438 buffer, conditions which result in negligible titre loss over repeated freeze-thaw cycles, or periods of years^6,8,19^.

24 h after infection cells were lysed with 1% Triton X-100 (Sigma), lysed cells were transferred to an opaque white 96-well plate (Thermo, 165306) and luciferase activity was quantified using a Bright-Glo assay kit (Promega) and Clariostar instrument (BMG). The infectivity of unknown samples was found by interpolation on the standard curve.

Infectivity titration by hexon immunostaining was performed using HEK293A cells^20^. Virus was serially diluted in Dulbecco’s modified Eagle’s medium supplemented with 10% foetal calf serum. 50 μL of dilution was added per well of a 96 well plate containing sub-confluent HEK293A cells. After 24 h a further 50 μL media was added. After 48 h infectivity was assessed. Cells were fixed at 20 °C in methanol, before the plates were blocked with 1% bovine serum albumin (BSA) in phosphate-buffered saline (PBS). Adenovirus hexon protein was stained by addition of 50 μL per well primary mouse anti-hexon polyclonal antibody (Cell Biolabs Inc.), followed by 50 μL secondary anti-mouse immunoglobulin horseradish peroxidise (HRP) conjugated antibody. Staining was developed by addition of 3,3’ diaminobenzidine (DAB) substrate, and positive brown stained cells in the wells were counted using a plate reader (Autoimmun Diagnostika GmBH [AID]).

### Freeze drying

All formulations used in the study are listed in Supplementary Data, sheet 1. The excipient stock solution was prepared at ambient temperature and used within 1 week. The stock solution was prepared at 10/9-fold desired final concentration and mixed with virus at 9:1 volume ratio.

Drying was performed either in microplates (Greiner, catalogue number 655901) or Type I glass vials with a nominal volume of 2 mL (Adelphi). To minimise the edge effect during drying of plates, samples were surrounded with water-filled plates.

Freeze-drying was performed using Virtis Advantage Plus or Lyostar 3 instruments (both from Scientific Products). All freeze-drying cycle parameters used in the study are listed in Supplementary Data Sheet 2.

Caps (Micronic, catalogue number MP53099) were placed on microplates prior to drying, and inserted using the dryer’s stoppering function after cycle completion.

Vials were sealed with bromobutyl rubber stoppers (Adelphi). For experiments performed using the Advantage Plus dryer, stoppers were inserted using the dryer’s stoppering function. For experiments performed using the Lyostar 3 instrument, stoppers were inserted by hand after removal from the instrument unless otherwise stated. Stoppered vials were hand crimped.

Freeze-dried cakes were scored against a rating scale from 0.0 (worst) to 1.0 (best), by an observer blinded to the formulation (Supplementary Fig. 2).

### Storage and reconstitution

Samples were stored in temperature-monitored environments at the stated temperatures +/− 2 °C.

Freeze-dried cakes were reconstituted in either phosphate-buffered saline or water, to the original (pre-drying) volume unless otherwise stated. Samples were thoroughly mixed to ensure complete resuspension and dissolution.

### Product characterisation

Dynamic Light Scattering (DLS) was performed on reconstituted samples using a Zetasizer Ultra instrument (Malvern Panalytical, UK). The viscosity of each formulation was corrected using 100 nm latex beads based on the Stokes-Einstein equation.

Residual moisture was measured using a coulometric Karl Fischer instrument (Metrohm) in accordance with the manufacturer’s instructions.

For Modulated Differential Scanning Calorimetry (mDSC) of both frozen liquid and dried samples, the material was loaded into a pre-weighted Tzero pan with Tzero hermetic lid (TA Instruments). The sealed pan was weighed again to calculate the net sample weight. mDSC was performed on a Q2000 DSC (TA Instruments, Elstree, Herts, UK) using the sample pan together with an empty reference pan. Heat-cool modulation ( ± 0.636 °C) was applied for all the samples with 60 s modulation period. The cycle involved equilibration at −70 °C, isothermal hold for 5 min, and ramping at 1 °C/min to 100 °C. The calorimetric data were analysed by Universal Analysis 2000 software (TA Instruments) with T_g_’ (for frozen liquid), T_g_ (for dried solids), and in some cases melting temperature (*T*_melt_) (for frozen liquid) reported. An endothermic integration was performed for the melting event. The melting onset temperature was obtained by linearly extrapolating the baseline 10–15 °C after the end of melting.

### Analysis

Multi-factorial experiments were analysed using DesignExpert 13 software (StatEase). Selection of model followed the procedure recommended in the software user guide. Data transformations were selected according to the minima on Box-Cox plots of each data set. User-guide-recommended diagnostics were applied to confirm appropriateness of the fitted models. The selection of optimal formulations was performed using the software’s maximisation function. For data sets including both titre change and cake score as responses, equal weights were assigned to each.

Graphs were prepared using Prism 9.0 (GraphPad) or DesignExpert.

### Reporting summary

Further information on research design is available in the Nature Research Reporting Summary linked to this article.

## Supplementary information

Supplementary Information

Supplementary Data

REPORTING SUMMARY

## Data Availability

The data that support the findings of this study are available from the corresponding author upon reasonable request.
