# Supplementary material for: Lyophilization to enable distribution of ChAdOx1 and ChAdOx2 adenovirus-vectored vaccines without refrigeration
Source: NPJ Vaccines. 2023 Jun 5;8:85. doi: 10.1038/s41541-023-00674-2 (PMC10240132; doi:10.1038/s41541-023-00674-2)
Supplement: Supplementary file 1 — Supplementary Information [file 41541_2023_674_MOESM1_ESM.pdf]

Supplementary Information

Supplementary Figure 1: Performance of luciferase-based infectivity assay

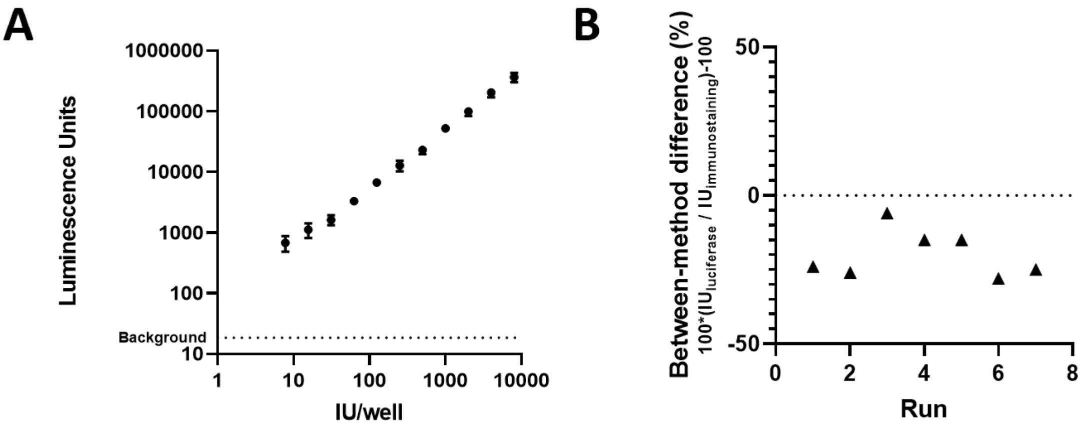

(A) Standard curve linearity over 1000-fold range. Points indicate mean of 2 replicate wells. Error bars indicate range.

(B) Run chart showing seven independent luciferase-based infectivity assays of aliquots of a single sample, with comparison against titre of that sample based upon the gold standard hexon immunostaining assay. The mean between-method difference of -20% is within the margin of error of the immunostaining assay itself, in our laboratory. The inter-assay coefficient of variation was 9%.

Supplementary Figure 2: Cake scoring scale

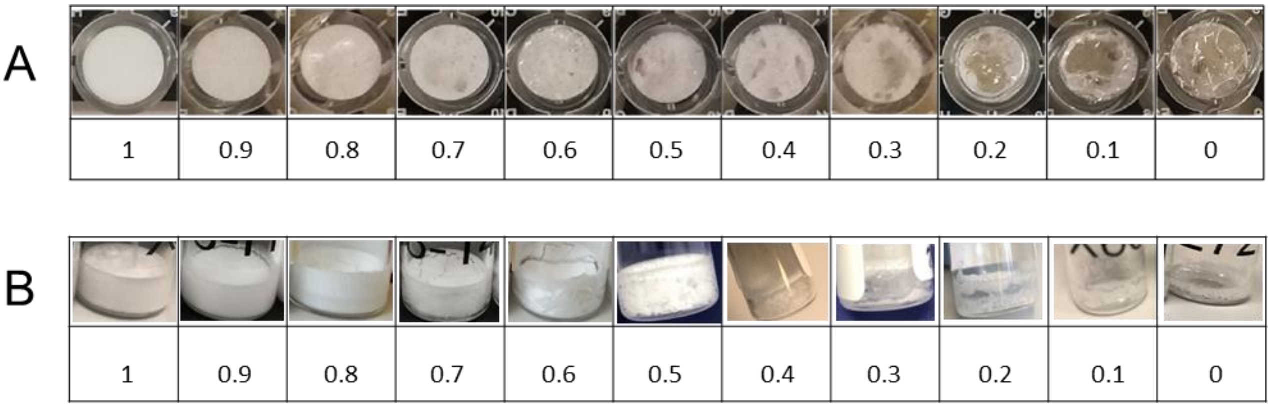

Cakes were scored visually from worst (0) to best (1) by comparison to the above scales for cakes viewed from the underside of plates (A) or in vials (B). In experiments with multiple formulations, the scorer was blinded to the formulation.

Supplementary Figure 3: Addition of dextran, PVA, glycine, or serine

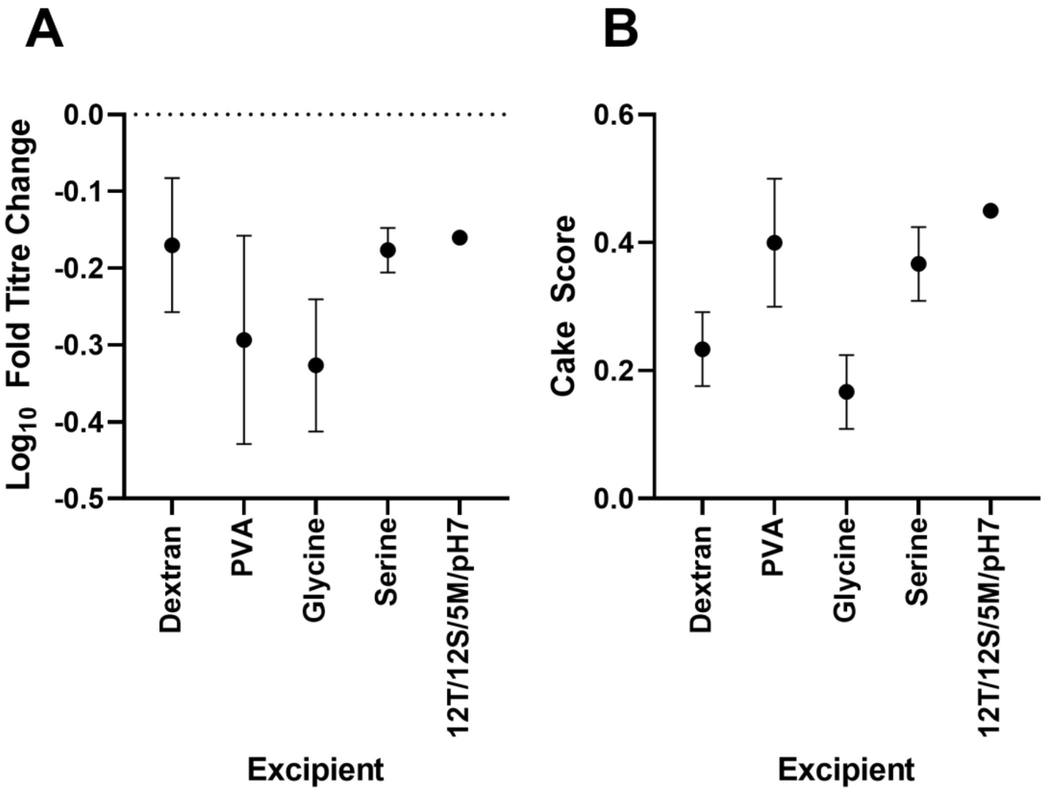

Mean titre change (A) and cake score (B) after addition of excipients to a base formulation of 15% trehalose, 15% sucrose, 5% mannitol and 10mM histidine pH 7. Excipients were either 1% dextran, 1% PVA, 0.25M glycine or 0.1M serine. Points indicate mean of three replicate wells. Error bars indicate range. A similar formulation with no added excipients and slightly lower sugar concentration is shown on the right for comparison (12% trehalose, 12% sucrose, 5% mannitol, 10mM histidine pH 7). Samples were dried using cycle A (Supplementary Table 2) in Lyostar 3.

Supplementary Figure 4: Poorly-stabilising formulations result in product aggregation

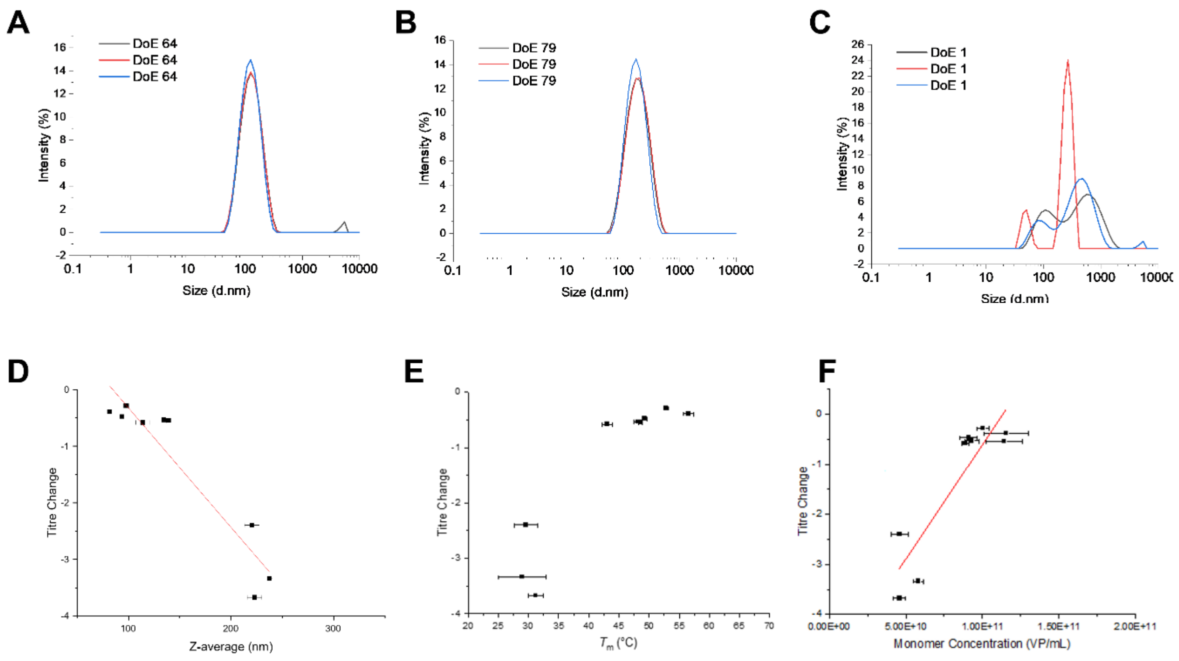

Nine formulations were selected, spanning the range of stabilization performance observed in the screening (three good, three intermediate and three poor). Virus was lyophilized in each formulation then reconstituted, and biophysical measurements were made on the reconstituted solution.

(A)-(C) DLS intensity distributions of reconstituted formulations with (A)good, (B) intermediate and (C) poor performance in maintenance of product infectivity.

(D-F) Relationships of biophysical parameters with infectivity preservation (data taken from second screening experiment, as shown in Figure 2). Points indicate mean measurements and error bars indicate standard error of the mean of 3 replicate samplings from a single reconstitute well

(D) Particle size (represented as DLS Z-average)

(E) Protein melting temperature (T_m, inferred from change in barycentric mean of intrinsic fluorescence during a temperature ramp)

(F) Concentration of genome-containing virus particles, derived from UV spectrophotometry of SDS-treated solutions.

Supplementary Figure 5: Effect of cycle modification on product stability and cake morphology

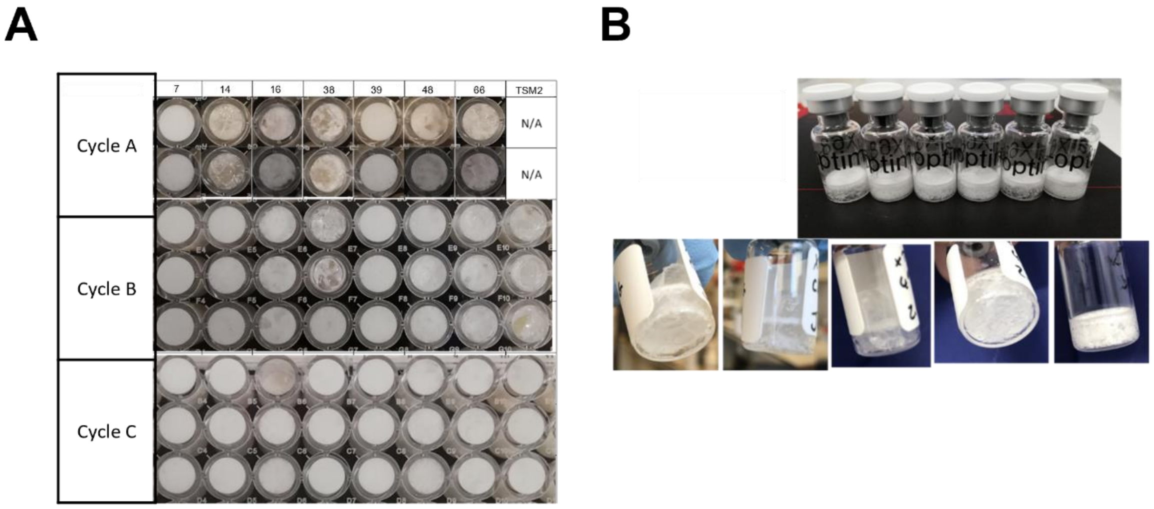

(A) Cake morphology in plates with and without annealing +/- enhanced primary drying.
(B) Cake morphology in vials after lyophilization with (top) and without (bottom) annealing

Supplementary Figure 6: Cake appearance and stability after slow-ramp drying with varying fill depths

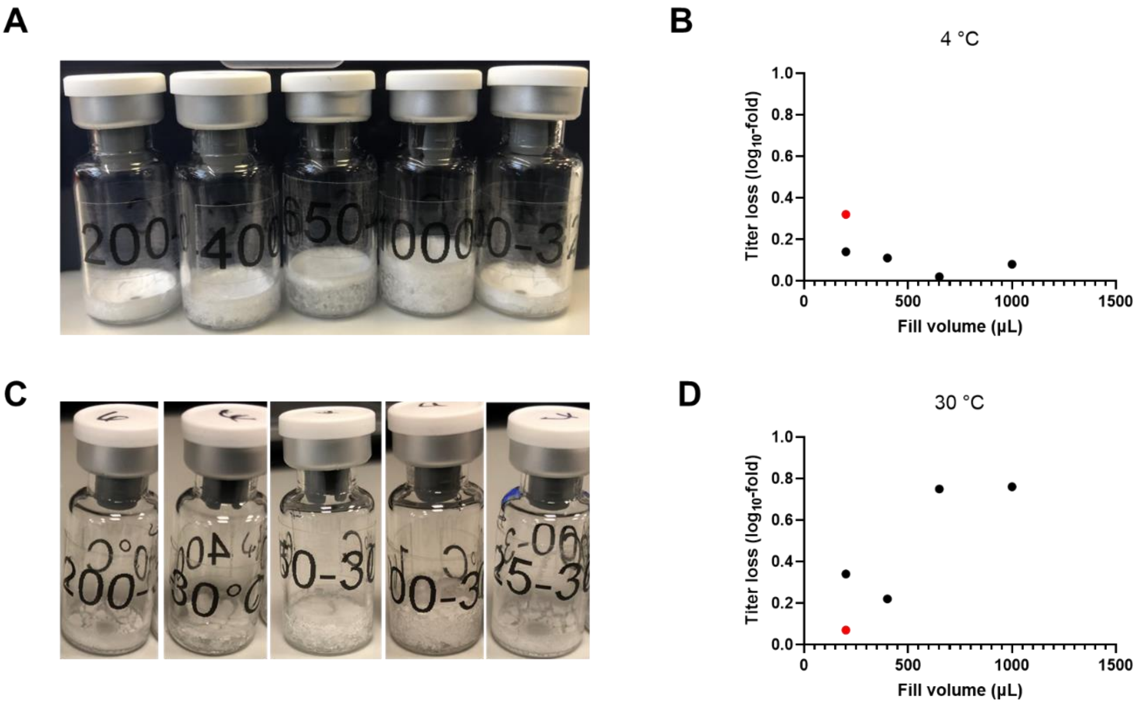

Vials were filled with a range of volumes formulation TSM2 containing ChAdOx1 luciferase at $1x10^{11}$ VP/mL, or with 200 µL at $3.25x10^{11}$ VP/mL, and subjected to lyophilization using a cycle combining annealing with a slow temperature ramp during drying (Cycle E) in the Lyostar 3.

(A) Cake appearance of representative vials and (B) titre loss as compared to pre-lyophilization, both after 50 days at 4 °C. Cake appearance was essentially unchanged from that immediately after drying. The red point shows the titre loss of the 200µl vial loaded at $3.25x10^{10}$ VP/ml.

(C) Cake appearance of representative vials and (D) titre loss as compared to pre-lyophilization, both after 50 days at 30 °C. The red point shows the titre loss of the 200µl vial loaded at $3.25x10^{10}$ VP/ml.

Supplementary Figure 7: Effect of drying cycle modification and trehalose / sucrose ratio on cake score

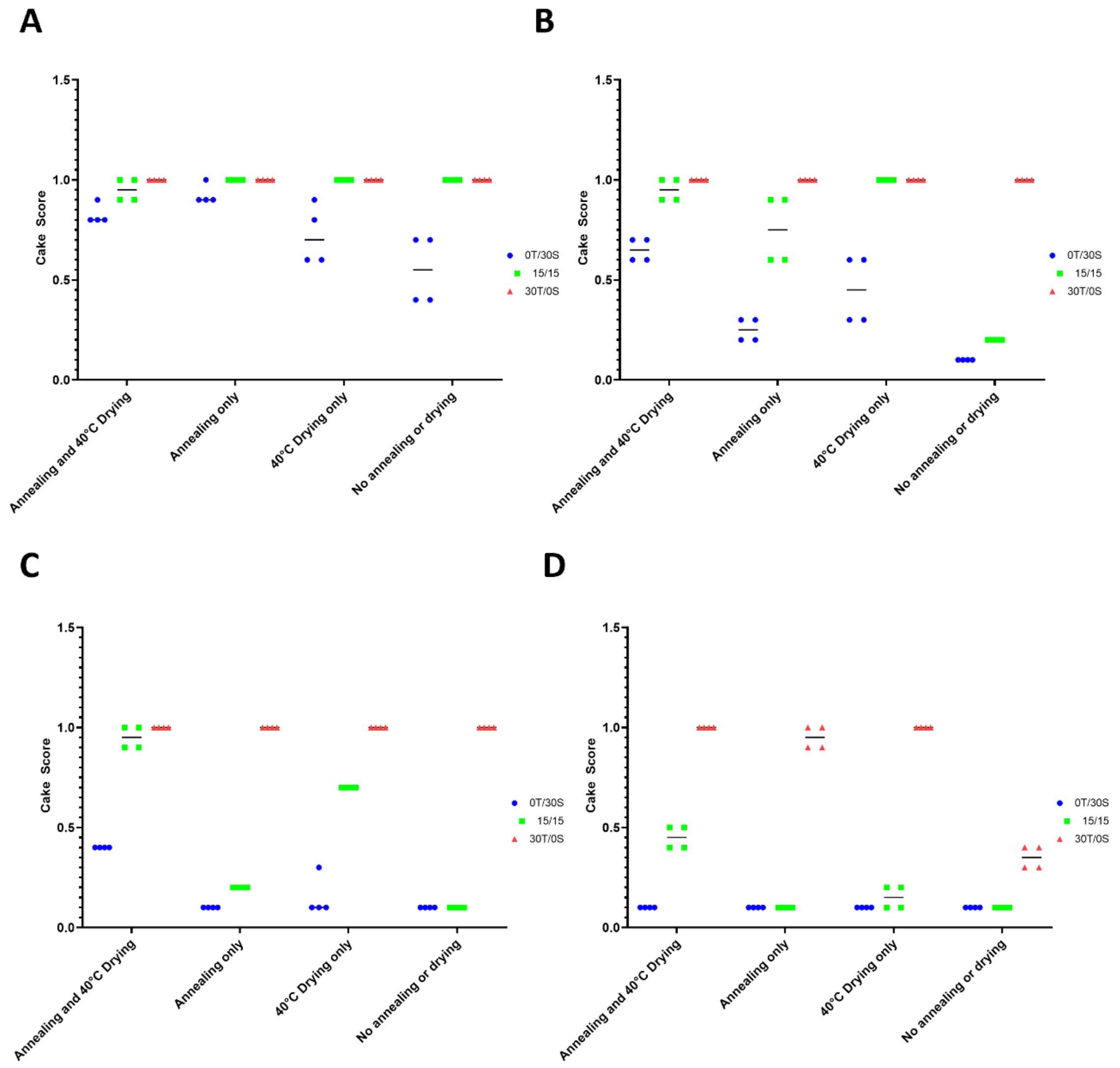

Effect of drying cycle modification and trehalose / sucrose ratio upon cake appearance within the experiment for which potency stability is reported in Figure 4. Panels show cake score after one month at 4°C (A), 30°C (B), 37°C (C) and 45°C (D), illustrating that cycles incorporating annealing and/or drying tended to have better cake stability at high temperatures.

Supplementary Table 1

Details of formulations used in study – please see Excel file.

Supplementary Table 2

Details of lyophilization cycles used in study – please see Excel file
